# Different effect of hydroxyurea and phlebotomy on prevention of arterial and venous thrombosis in Polycythemia Vera

**DOI:** 10.1038/s41408-018-0161-9

**Published:** 2018-11-26

**Authors:** Tiziano Barbui, Valerio De Stefano, Arianna Ghirardi, Arianna Masciulli, Guido Finazzi, Alessandro M. Vannucchi

**Affiliations:** 1 FROM Research Foundation, ASST Papa Giovanni XXIII, Bergamo, Italy; 2 Institute of Hematology, Catholic University, Roma, Italy; 3 IRCCS Policlinico Gemelli Foundation, Roma, Italy; 4 USC Hematology, ASST Papa Giovanni XXIII, Bergamo, Italy; 5 Department of Experimental and Clinical Medicine, Center of Research and Innovation of Myeloproliferative neoplasms (CRIMM), AOU Careggi, University of Florence, Florence, Italy

Clonal proliferation of hematopoietic precursors leading to progressive expansion of myeloid cells with a predominant increase of red cells characterizes the hematological phenotype of Polycythemia Vera (PV). The resulting blood hyperviscosity is a major determinant of vascular complications which severely impact on morbidity and mortality of these patients^1^. Aggressive maintenance of a target hematocrit level lower than 45% with phlebotomy (PHL), either alone or associated with cytoreductive drugs, and low-dose aspirin have been shown to reduce the thrombosis rate in the randomized controlled CYTO-PV^2^ and European Collaborative Low-dose Aspirin Polycythemia Vera (ECLAP)^3^ clinical trials, respectively, and are recommended therapies in the clinical practice^4,5^.

In patients at high-risk because of age and/or history of thrombosis, hydroxyurea (HU) is the recommended front-line cytoreductive drug based on a small observational study (PVSG protocol 08), in which 51 HU-treated patients experienced a lower incidence of thrombosis compared with historical controls managed with PHL^6^.

More recently, interferon (IFN)-α and ruxolitinib (a JAK1 and JAK2 inhibitor) have been introduced into the therapeutic armamentarium for patients with PV, yet in the absence of controlled evidence of superiority over HU as regards prevention of thrombosis, and a relative lack of information about safety over long-term use for ruxolitinib^7^.

The recommendation that HU should be a first line therapy has been criticized since no solid demonstration of its efficacy to prevent thrombosis or prolong survival has been produced so far^7^. Moreover, the concern that HU may increase the risk of leukemic transformation led to suggest therapeutic PHL as the only first line therapy, irrespective of patient risk category^8^. Recently, we documented an advantage of HU over PHL in a cohort of 1042 patients with PV included in the ECLAP trial consistently significant with respect to the proportion of fatal/non-fatal CV events (13.2% vs. 7.9% in PHL vs. HU groups, respectively, *p* = 0.006) and myelofibrosis transformation, that was more frequent in patients treated with PHL only^9^.

In the present work we investigated more thoroughly the same cohort of PV patients to determine whether cytoreduction therapy with HU, in comparison with therapeutic PHL, was differently effective in protecting from arterial and venous thrombosis. The rationale of this analysis, approved by our IRB, is supported by the following considerations: (i) the two vascular complications are biologically different processes with distinct physiopathology supporting that arterial and venous events are separate entities with specific risk factors that require careful evaluation and management^10^. (ii) HU may predominantly exert its antithrombotic property by reducing the cellular components of thrombi formation, including leukocytes, and (iii) leukocytosis is associated more with arterial than venous thrombosis in PV^1^.

We selected patients who, during the follow-up, had received only PHL or HU to maintain the HCT level < 45%. To assure comparability, we conducted a 1:2 Propensity Score (PS) matching analysis^11^ by forming matched sets of 1 PHL and up to 2 randomly sampled HU-treated subjects who shared a similar values of PS (estimated by regressing exposure to PHL only conditionally on the baseline covariates reported in Table 1). The two groups (PHL *n* = 317 and HU *n* = 634) were well balanced for the parameters included in the PS (overall balance test: *p* = 0.673). Over a comparable treatment period with PHL (median: 25.8 months) and HU (median: 24.0 months), we documented a significant advantage of HU over PHL with respect to the proportion of non-fatal arterial thrombosis (6.3% vs. 2.4% in PHL vs. HU groups, respectively, *p* = 0.002), while HU did not result more effective than PHL in reduction of unprovoked venous thromboembolism (*p* = 0.574) (Table 1). The rate of arterial thrombosis was threefold lower in patients on HU vs. PHL (2.62 vs. 0.84 per 100 person-years (PY) in PHL vs. HU groups, respectively, *p* = 0.001).

**Table 1:** Baseline characteristics and thrombosis during follow-up in 1:2 propensity-score-matched patients with Polycythemia Vera treated with phlebotomy only or hydroxyurea

	1:2 random-sample^a^ matched cohort (*n* = 951)		
	PHL (*n* = 317)	HU (*n* = 634)	*p*
Baseline characteristics			
Age at enrolment ≥ 60, *n* (%)	172 (54.3%)	346 (54.6%)	0.272
Male*, n* (%)	221 (69.7%)	463 (73.0%)	0.195
Years from diagnosis of PV to enrolment ≥ 5*, n* (%)	92 (29.0%)	161 (25.4%)	0.148
Prior thrombosis*, n* (%)	104 (32.8%)	221 (34.9%)	0.915
High risk*, n* (%)	202 (63.7%)	421 (66.4%)	0.127
Active smoking*, n* (%)	65 (20.5%)	95 (15.0%)	0.901
Hypertension*, n* (%)	131 (41.3%)	229 (36.1%)	0.841
Diabetes mellitus*, n* (%)	25 (7.9%)	41 (6.5%)	0.482
Aspirin use*, n* (%)	177 (55.8%)	359 (56.6%)	0.393
Oral anticoagulant*, n* (%)	19 (6.0%)	36 (5.7%)	0.057
BMI categories*, n* (%)			
Underweight /normal range	142 (44.8%)	279 (44.0%)	0.670
Overweight	139 (43.8%)	293 (46.2%)	
Obese	36 (11.4%)	62 (9.8%)	
Follow-up			
Median total follow-up (IQR), months	29.9 (15.1, 41.0)	34.7 (24.1, 45.3)	0.001
Median treatment duration (IQR), months	25.8 (12.7, 37.3)	24.0 (12.0, 36.0)	0.696
Arterial thrombosis	20 (6.3%)	15 (2.4%)	0.002
IR/100 PY (95% CI)	2.62 (1.69, 4.05)	0.84 (0.51, 1.39)	0.001
Myocardial infarction	1	5	
Stroke	8	2	
Transient ischemic attack	7	8	
Peripheral arterial thrombosis	4	0	
Venous thrombosis	10 (3.2%)	16 (2.5%)	0.574
IR/100 PY (95% CI)	1.29 (0.69, 2.40)	0.90 (0.55, 1.47)	0.380
Deep vein thrombosis	4	9	
Superficial vein thrombosis	6	7	

*PHL* Phlebotomy, *HU* hydroxyurea, *IQR* interquartile range, *IR* incidence rate, *PY* person-years

^a^Two randomly sampled HU patients: 1 PHL patient in each matched subset. Matching was done using the nearest neighbor method with replacement and with caliper of width equal to 0.2 of the pooled standard deviation of the logit of PS

**Fig 1.**
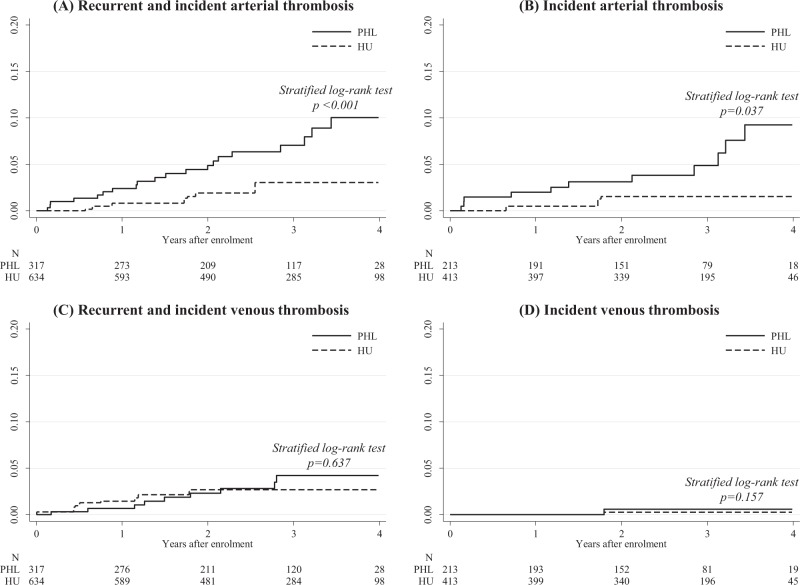
Cumulative incidence of thrombosis in the patient cohort. Cumulative incidence of arterial (a) and venous (c) thrombosis in the total matched cohort of patients with or without prior thrombosis, and cumulative incidence of arterial (b) and venous (d) thrombosis in patients who were thrombosis-free at study entry

The cumulative incidence curves for arterial thrombosis (Fig. 1a, b) showed that the different effect of HU and PHL was observed for both patients with or without prior history of thrombosis. This findings were not confirmed for the incidence of venous thrombosis (Fig. 1c, d).

The results of this study comparing two well-balanced cohorts of PV patients by using a propensity score matching, should be interpreted as a semi-experimental prospective trial and although the findings of current analysis are possibly exposed to intrinsic limitations, the rigorous application of 1:2 matching (that included relevant variables as reported in the Table 1) should reduce the limits of this retrospective analysis.

To our knowledge, this is the first study documenting the greater antithrombotic protection of HU over PHL against arterial thrombosis while the two treatments produce similar results in the protection from venous thrombosis. This observation deserves to be confirmed with studies that have a larger number of venous events including splanchnic thrombosis separately from the common deep vein thrombosis. One possible explanation for these findings is that HU reduces the abnormalities of MPN clone-derived blood cells, such as leukocytosis and consequently the interaction with platelets and vascular endothelium which lead to an inflammatory state reduces the inflammatory state we know involved in the pathogenesis of thrombosis^1^. Moreover, the antithrombotic effect of this drug may recognize additional mechanisms of action besides pan-myelosuppression, including qualitative changes in leukocytes, decreased expression of endothelial adhesion molecules, and enhanced nitric oxide (NO) generation^12^. These processes are more involved in arterial thrombosis, while the changes in blood flow due to hyperviscosity are predominantly associated with venous thrombosis, providing an explanation for the comparable efficacy of PHL and HU on the background of similar control of HCT levels < 45%. However, it should be underscored that the rate of both arterial and venous thrombosis remains high, also in low-risk PV (rate 2.5 per 100 PY^2^) compared to the normal population (0.25 per 100 PY for ischemic stroke and acute myocardial infarction, and 0.1–0.2 per 100 PY for venous thromboembolism)^13^, suggesting the need of further therapeutic proposals.
